# Refinement of a Live Attenuated *Salmonella enterica* Serovar Newport Vaccine with Improved Safety

**DOI:** 10.3390/vaccines9010057

**Published:** 2021-01-16

**Authors:** Shamima Nasrin, Fabien J. Fuche, Khandra T. Sears, Jennifer A. Jones, Myron M. Levine, Raphael Simon, Sharon M. Tennant

**Affiliations:** Center for Vaccine Development and Global Health, Department of Medicine, University of Maryland School of Medicine, Baltimore, MD 21201, USA; snasrin@som.umaryland.edu (S.N.); fabien.fuche@gmail.com (F.J.F.); ksears@som.umaryland.edu (K.T.S.); jennifer.jones@crisphealth.org (J.A.J.); mlevine@som.umaryland.edu (M.M.L.); rsimon@som.umaryland.edu (R.S.)

**Keywords:** *Salmonella*, vaccine, live, attenuated, Newport, Muenchen

## Abstract

Non-typhoidal *Salmonella* (NTS) is a major cause of gastroenteritis and is responsible for approximately 93 million cases annually. In healthy individuals, gastroenteritis caused by NTS is usually self-limiting, however, NTS can cause severe invasive disease in immunocompromised patients. Very little research has been directed towards development of vaccines against *Salmonella* serogroups O:6,7 or O:8. We have constructed a live attenuated serogroup O:8 vaccine, CVD 1979, by deleting *guaBA*, *htrA*, and *aroA* from the genome of *S*. Newport. We have shown that the candidate vaccine is well tolerated in mice and elicits serum immunoglobulin G (IgG) antibodies against core O-polysaccharide (COPS) when administered orally. Immunized mice were challenged intraperitoneally with wild-type *S*. Newport and bacterial burden in the liver and spleen was found to be significantly reduced in the livers of immunized mice compared to control mice. We also observed moderate vaccine efficacy (45%) against lethal challenge with the serogroup O:8 serovar, *S*. Muenchen, but low vaccine efficacy (28%) following lethal challenge with a serogroup O:6,7 serovar, *S*. Virchow. In vitro, we have shown that antibodies generated by CVD 1979 only recognize lipopolysaccharide (LPS) from serogroup O:8 but not serogroup O:6,7 serovars, and that they mediate opsonophagocytic antibody (OPA) activity against serogroup O:8 but not serogroup O:6,7 serovars. We also showed that OPA activity can be blocked by pre-incubating the antisera with serogroup O:8 lipopolysaccharide. Taken together, our data demonstrate that we have constructed a well-tolerated, effective live attenuated *S*. Newport vaccine which elicits functional antibodies against serogroup O:8 but not O:6,7 serovars.

## 1. Introduction

Non-typhoidal *Salmonella* (NTS) are a major cause of gastroenteritis worldwide and are estimated to cause 93.8 million cases of illness globally each year, with 155,000 deaths [1]. A substantial proportion of cases are foodborne [1] and, in the United States, NTS are responsible for multiple foodborne outbreaks each year [2]. In most cases, NTS cause self-limiting gastroenteritis that does not require any therapeutic intervention, however, they can cause severe invasive infections in children under 5 years of age living in low resource countries, the elderly, and immunocompromised patients [3,4,5,6,7].

Serogroup O:4 (B), which includes *S.* Typhimurium, and serogroup O:9 (D), which includes *S.* Enteritidis, are the most common serogroups accounting for approximately 50% of all reported human NTS isolates in the United States [8]. Serogroups O:6,7 (C_1_) and O:8 (C_2_–C_3_), are becoming more prevalent in humans and animals [8]. Together, O:4, O:6,7, O:8, and O:9, are the most commonly reported serogroups from human cases in the United States and European Union [9,10]. In Asia and Africa, the NTS burden is underestimated due to lack of surveillance, yet serogroups O:6,7 and O:8 are the second (27.3% of NTS cases) and third (19.5% of NTS cases) leading causes of *Salmonella* infections in humans, respectively [8]. In countries like Taiwan and Ethiopia, certain serovars (e.g., *S*. Choleraesuis and *S*. Concord) from group O:6,7 are more frequently isolated from blood and have been found to be invasive in 30–56% of cases [11,12,13]. *S*. Choleraesuis is known to be associated with severe invasive infections in humans including bacteremia, extraintestinal localized infections in multiple organs, and more likely to require hospitalization [8,13]. *Salmonella* Newport (O:8), is one of the most common etiological agents of gastroenteritis in the United States [8,9,14,15]. *S*. Newport is also associated with severe invasive infections in humans [13]. The incidence of serovar Newport has increased during the period 1996–2011 in the USA [9].

Little research has been performed on *S*. Newport to date. Most NTS vaccine development has focused on *S*. Typhimurium and *S*. Enteritidis and several live attenuated and conjugate vaccines against these serovars have shown promising vaccine efficacy in pre-clinical studies [16,17]. Our group has developed both glycoconjugate [18] and live attenuated [19] *S*. Newport vaccines that have shown protection against *S*. Newport infection in mouse models. We previously constructed the *S*. Newport live attenuated vaccine CVD 1966 by deleting the *guaBA* (encodes guanine biosynthesis) and *htrA* (encodes a heat shock response protein) genes which are known to have a strong attenuating effect in various *Salmonella* serovars (e.g., Typhimurium and Typhi) [20,21]. The candidate *S*. Newport vaccine, CVD 1966 (Δ*guaBA* Δ*htrA*) was attenuated, yet immunogenic in mice [19]. Following intraperitoneal immunization, CVD 1966 elicited robust immune responses against core-*O* polysaccharide (COPS), flagellin and outer membrane proteins and significantly reduced the bacterial burden in the liver and spleen of infected animals. However, a few animals experienced impaired balance following oral immunization with CVD 1966. Thus, we sought to improve the safety and tolerability of this *S*. Newport candidate vaccine by deleting an additional aromatic amino acid biosynthesis gene, *aroA*, which has previously been used to reduce the virulence of *S*. Typhimurium [22]. Deletion of genes in the aromatic amino acid biosynthesis pathway (e.g., *aroA*, *aroC*, and *aroD*) makes the bacteria auxotrophic for para-aminobenzoic acid (PABA) and 2,3-dihydrobenzoate and *aro* mutants are unable to scavenge sufficient PABA and dihydrobenzoate to replicate in mice [22]. Several live attenuated *Salmonella* Δ*aroA* vaccines have been developed for *S*. Typhimurium [23], *S*. Bovismorbificans [24], and *S*. Choleraesuis [25]; pre-clinical studies have shown that those vaccines elicit strong immune responses and protect animals against lethal challenge.

The goal of the present study was to refine our *S*. Newport vaccine, CVD 1966 (Δ*guaBA* Δ*htrA*) by incorporating an additional deletion in *aroA* to develop a safe and effective live attenuated vaccine that can protect against infections caused by serovars of serogroup O:8 (C_2_–C_3_). Additionally, we examined efficacy of this vaccine in mice against heterologous challenge with *S*. Virchow, an O:6,7 (C_1_) serovar. We also investigated the specificity of vaccine-induced antibodies against O:6,7, and O:8 serovars and the opsonophagocytic antibody activity of anti-lipopolysaccharide (LPS) antibodies. We report herein the development and assessment of an oral live attenuated *S*. Newport candidate vaccine, CVD 1979.

## 2. Materials and Methods

### 2.1. Bacterial Strains, Plasmids, and Culture Conditions

The bacterial strains used in this study are listed in Table 1. Plasmids pKD13, pKD46, and pCP20 were used to delete genes from specific chromosomal loci. All *Salmonella* strains were maintained in animal-product-free Hy-Soy (HS) medium (10 g/L Soytone (Teknova, Hollister, CA), 5 g/L Hy-yest (Kerry BioScience, Beloit, WI, USA) and 5 g/L sodium chloride (American Bio, Natick, MA, USA)) at 37 °C. When needed, agar (American Bio) was added at 15 g/L. Medium was supplemented with guanine (0.005% (w/v) final concentration; Sigma–Aldrich, St. Louis, MO, USA) for mutants harboring *ΔguaBA* deletions. Carbenicillin (Corning, Glendale, AZ, USA) or kanamycin (Sigma–Aldrich) at a final concentration of 50 µg/mL were added when necessary.

### 2.2. Construction of S. Newport Mutants

*S*. Newport CVD 1966 was previously generated by Fuche et al. [19] derived from *S*. Newport Chile 361, an invasive clinical isolate, and harbors deletions in *guaBA* and *htrA.* Mutants were constructed by deleting the *aroA* gene from the chromosome of wild-type (WT) *Salmonella* Newport Chile 361 [26] and CVD 1966 using lambda red-mediated homologous recombination [29]. The entire open reading frame (ORF) was deleted and the deletion was confirmed genotypically by PCR and sequencing using primers that were located at least 500 bp upstream and downstream of the targeted gene. Primers used to construct these mutants in this study are aroAmutF (GTCCATCCTCGACTACACCG), aroAmutRkan (GAAGCAGCTCCAGCCTACACTAAAAACCCCACAGACTGGC), aroAmutFkan (GGTCGACGGATCCCCGGAATAAGTCTTCTGTTGCGCCAGT), aroAmutR (TGCGTTGATATCGCTGGTCA).

### 2.3. 50% Lethal Dose (LD_50_) Analysis

All animal procedures were approved by the Institutional Animal Care and Use Committee of the University of Maryland School of Medicine. Work was performed under protocol #0816019 approved on 11 October 2016 and #0619004 approved on 25 July 2019. We evaluated the virulence of the *aroA* mutant by determining the 50% lethal dose (LD_50_) in mice. The mutant strain was streaked onto HS medium and grown for 18–20 h at 37 °C. Bacterial colonies were then resuspended in sterile phosphate buffered saline (PBS) to an optical density (OD) at 600 nm of 1.25 (1 × 10^9^ colony-forming units (CFU)/mL). Groups of five 6 to 8-week-old female BALB/c (Charles River Laboratories, Wilmington, MA) mice were infected intraperitoneally (i.p.) with six ten-fold dilutions of (1 × 10^4^ to 1 × 10^9^ CFU) of the *S*. Newport Δ*aroA* mutant. Viable counts were performed to verify the number of bacteria administered to mice. Following infection, mice were weighed and monitored daily for 28 days for signs of illness and weight loss. Any mouse that lost more than 20% of its body weight as compared to its weight at the time of infection or that showed signs of extreme morbidity (e.g., shallow breathing or hunched posture) was euthanized and scored as a death. The LD_50_ of the strain was calculated by linear regression analysis.

### 2.4. Mouse Immunization and Protection against Challenge

The 6 to 8-week-old female BALB/c mice (Charles River Laboratories) were immunized per orally with 100 μL PBS containing 10^9^ CFU of live attenuated *S*. Newport vaccine CVD 1979 or PBS alone, three times with three weeks between each immunization, and monitored for clinical signs. Then, four weeks after the last immunization mice were challenged i.p. with WT *Salmonella* strains to assess the homologous and heterologous protection elicited by the live attenuated *S*. Newport vaccine CVD 1979. For homologous protection experiments, CVD 1979-vaccinated or PBS-treated mice (n = 10) were challenged with low (4 × 10^5^ CFU) and high (1 × 10^7^ CFU) doses of *S*. Newport Chile 361. Then, two days post-infection, mice were euthanized, and their liver and spleen were collected. Organs were homogenized, and dilutions were spread onto HS plates to determine the CFU/g. To determine the heterologous protection elicited by the live attenuated *S*. Newport CVD 1979 vaccine, vaccinated or PBS-treated mice were challenged i.p. (n = 20) with an LD_100_ of *S*. Muenchen ATCC 8344 (3 × 10^7^ CFU) or *S*. Virchow Q23 (5 × 10^7^ CFU). After challenge, mice were monitored for 28 days for weight loss and mortality as described for the 50% lethal dose analysis.

### 2.5. Measurement of Serum Antibodies

One day before each vaccination (day—1, 20, 42) or challenge (day 66), blood was collected from each mouse to determine the levels of serum IgG against core-*O*-polysaccharide (COPS) by enzyme-linked immunosorbent assay (ELISA) as described previously [19]. *S*. Newport COPS was purified as previously described [19]. Briefly, medium-binding 96-well microtiter plates (Greiner Bio-One, Monroe, NC, USA) were coated with 100 μL/well of *S*. Newport COPS antigen at a concentration of 5 μg/mL. COPS was diluted in carbonate-bicarbonate buffer (pH 9.6) and coated plates were incubated overnight at 4 °C. After incubation, plates were washed 6 times with 0.05% Tween 20 in PBS (PBST) and blocked with 200 μL/well of 10% non-fat dry milk (NFDM) in PBS for 1 h at 37 °C. Individual mouse serum was diluted in 10% NFDM in PBST and was tested in duplicate wells. Plates were incubated at 37 °C for 1 h then washed 6 times with PBST. Horse radish peroxidase (HRP)-conjugated goat anti-mouse IgG antibody (Bio-Rad Laboratories, Carlsbad, CA) secondary antibody was used according to the manufacturer’s instructions. Following six washes with PBST, antibody binding was detected using the TMB Microwell Peroxidase Substrate system (KPL, Gaithersburg, MD, USA). The ELISA titers (ELISA units (EU) per milliliter for antigen-specific antibodies) were calculated by linear regression analysis. Seroconversion was defined as a four-fold increase in titer after immunization, compared to pre-immunization titers.

### 2.6. Lipopolysaccharide (LPS) and Whole Cell Lysate Extractions

Lipopolysaccharide (LPS) and whole cell lysates were extracted from an equal number of bacterial cells as described [18]. LPS and crude proteins from five different serovars, *S*. Virchow Q23, *S*. Choleraesuis var Kunzendorf CDC 06-0894, *S*. Newport Chile 361, *S*. Muenchen ATCC 8344, *S*. Hadar 700093 and *S*. Newport Δ*rfaL* were separated on a 4–12% NuPAGE^®^ Bis-Tris acrylamide gel (Thermo Fisher Scientific, Waltham, MA, USA). LPS was visualized by staining the gel with Pro-Q Emerald 300 LPS Gel Stain (Molecular Probes, Eugene, OR, USA) per the manufacturer’s instructions and proteins were stained using the gel-code blue Coomassie reagent. Both LPS and proteins were visualized with a ChemiDoc™ MP instrument using Image Lab 5.1 software (BioRad Laboratories, Hercules, CA, USA). Specificity of the anti-COPS antibodies to LPS and whole cell lysates of *Salmonella* serovars were determined by immunoblotting. LPS was transferred onto a polyvinylidene fluoride (PVDF) membrane (BioRad Laboratories, Hercules, CA, USA) using a mini Trans-Blot Electrophoretic Cell system (BioRad) at 35 V overnight at 4 °C. Proteins were transferred onto the PVDF membrane by the Trans-Blot Turbo Transfer system (BioRad) at 1.3 A 25 V for 7 min based on the manufacturer’s instructions. PVDF membranes were then blocked with 5% bovine serum albumin (BSA) in tris-buffered saline containing Tween-20 (TBST) for 2 h at room temperature and washed 3 times with TBST. Following washes, membranes were probed with pooled sera collected from animals immunized with live attenuated *S*. Newport vaccine overnight at 4 °C. Bound antibodies were detected by incubating the blots with HRP-conjugated goat anti-mouse IgG for 1 h at room temperature and developed with Clarity ECL substrate (BioRad).

### 2.7. Opsonophagocytic Uptake by Macrophages

Mouse macrophage J774A.1 (hereafter referred to as J774) cells were used to perform an opsonophagocytic antibody activity assay as previously described [21]. Briefly, stationary phase cultures of *Salmonella* strains (~2.5 × 10^5^ CFU/50 μL) were incubated with 5 μL of heat-inactivated pre-immune mouse sera or sera collected from mice immunized with live *S*. Newport CVD 1979 vaccine (immune sera) for 30 min at room temperature to allow antibodies to opsonize the bacteria. After incubation, 10 μL of the opsonized bacteria were added to the 24-well tray seeded with 1 mL of 4.5 × 10^5^ /well J774 cells (multiplicity of infection (MOI) of 0.11) and incubated for 45 min at 37 °C in the presence of 5% CO_2_. Intracellular bacteria were enumerated by viable counts and percent phagocytosis was calculated by dividing the number of bacteria that survived after the gentamicin treatment by the initial inoculum and multiplying by 100. Each strain was tested at least three times in duplicate wells. The contribution of anti-COPS antibodies to OPA activity was assessed by preincubating sera collected from pre-immunized and immunized mice with 200, 400, and 800 µg/mL concentrations of COPS for 30 min at room temperature. Following incubation, opsonophagocytic uptake of *S*. Newport Chile 361 was determined as described above.

### 2.8. Statistical Analysis

Data were analyzed and plotted using GraphPad Prism 7 Software (La Jolla, CA, USA) and were shown as mean ± standard error of the mean (SEM). ELISA data was analyzed by Tukey’s multiple comparisons test and the OPA data were analyzed by Student’s *t*-test. Survival curves between the vaccinated and control groups for each challenge strain were compared with log rank test and vaccine efficacy was analyzed by Fisher’s exact test. For data that was not normally distributed, two-tailed Mann–Whitney rank-sum test was used instead of Student’s *t*-test. A *p*-value below 0.05 was considered significant for each test.

## 3. Results

### 3.1. Construction of Salmonella Newport aroA Mutant and Refined Vaccine Strain

The *aroA* gene was deleted from *Salmonella* Newport Chile 361 and CVD 1966 strains using the lambda red-mediated homologous recombination system [29]. The *aroA* gene was deleted from *S*. Newport Chile 361 to construct a mutant, SNE-aroA, with a single gene deletion (Table 1). To construct the refined live attenuated vaccine CVD 1979, we deleted the *aroA* gene from the genome of *S*. Newport CVD 1966 which harbors mutations in *guaBA* and *htrA* [19] (Table 1). PCR and sequencing analysis showed that the entire *aroA* open reading frame had been deleted in SNE-aroA and *S*. Newport CVD 1979. We have previously shown that the double mutant, CVD 1966, is attenuated as compared to the parental *S*. Newport strain, however, we observed impaired balance in the mice following oral (but not intraperitoneal) immunization with CVD 1966. Therefore, in order to further attenuate the vaccine strain, we deleted an additional gene, *aroA*, to construct our refined candidate *S*. Newport vaccine strain, CVD 1979, which harbors mutations in *guaBA*, *htrA* and *aroA*.

### 3.2. Assessment of Attenuation of Strains in the Mouse Model

We previously determined that the optimal infection route for *S*. Newport Chile 361 in BALB/c mice was the intraperitoneal route and that the LD_50_ was 5 × 10^6^ CFU [19]. The LD_50_ of CVD 1966 was also determined previously and was found to be 6.1 × 10^8^ CFU [19]. In this study, we showed that the deletion of *aroA* from *S*. Newport Chile 361 had a strong attenuating effect in BALB/c mice; the i.p. LD_50_ was 1.4 × 10^8^ CFU which is 100-fold higher than that of the parental strain.

### 3.3. Immunogenicity Elicited by S. Newport CVD 1979 

**Figure 1:**
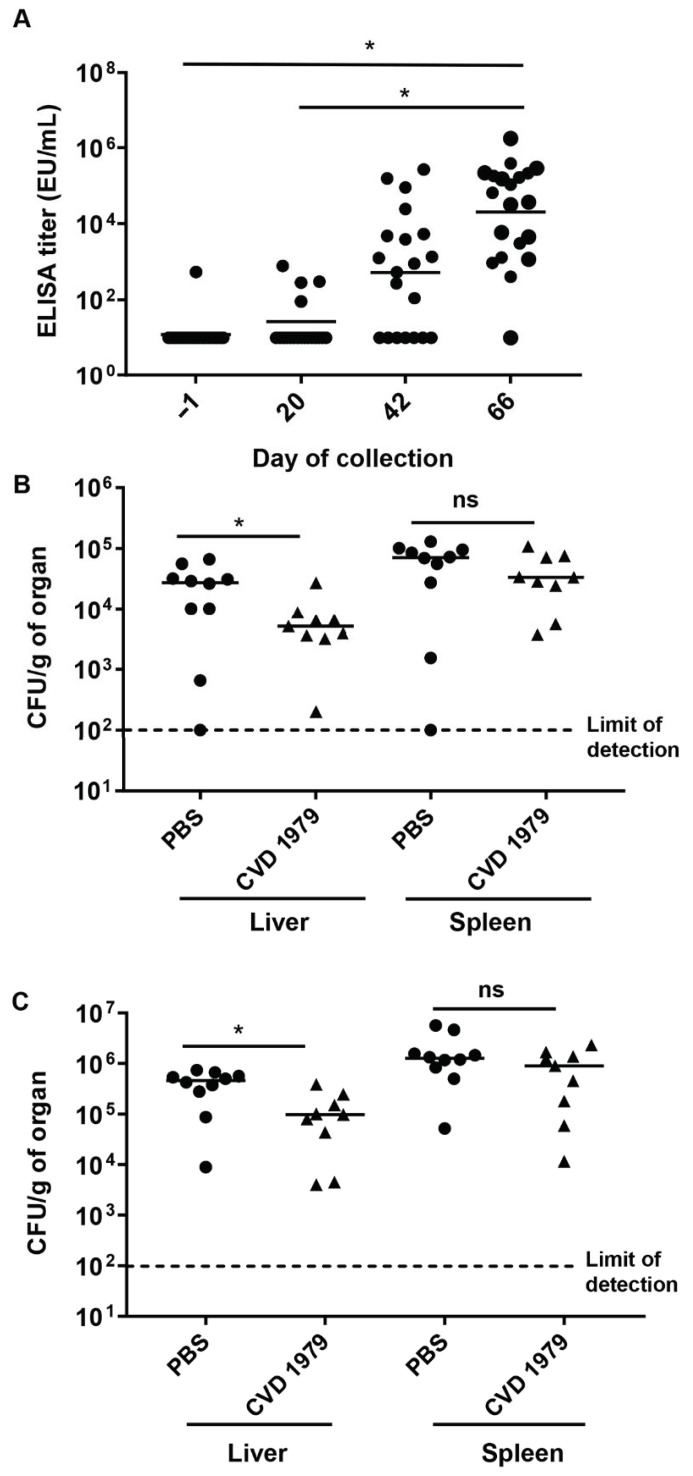
Immunogenicity and protective efficacy of live attenuated *Salmonella* Newport vaccine CVD 1979 in BALB/c mice. (**A**) Anti-LPS serum IgG titers in mice immunized orally with 3 doses of 10^9^ CFU of vaccine. Mice were bled before each immunization and challenge. Serum anti-LPS IgG antibody titer was determined for individual mice by ELISA. Black bars represent the geometric mean. Significant differences were determined using Tukey’s multiple comparisons test. (**B**) Bacterial burden in the liver and spleen of mice immunized with PBS or CVD 1979. One month after the 3rd immunization, mice were challenged intraperitoneally with a low dose (4.3 × 10^5^ CFU) of *S*. Newport Chile 361. (**C**) Bacterial burden in the liver and spleen of mice immunized with PBS or CVD 1979 and subsequently challenged with a high dose (1.4 × 10^7^ CFU) of *S*. Newport Chile 361. Two days post-infection, mice were euthanized, and their liver and spleen were collected. Organs were homogenized, and dilutions were spread onto HS plates for colony (CFU) counting. Bacterial loads are expressed as CFU per gram of organ. Black bars represent the median; significant difference was determined using two-tailed Mann–Whitney test. * *p* < 0.05; ns, not significant.

Mice were immunized with three doses of CVD 1979 or PBS three weeks apart via the oral route. Serum IgG titers against core-*O* polysaccharide (COPS) were measured prior to each immunization and before challenge. We found that the CVD 1979 vaccine was safe, there were no adverse side effects following immunization and animals that received CVD 1979 elicited strong immune responses following each immunization. The geometric mean titer (GMT) increased significantly after three doses of vaccine (Figure 1A) as compared to the first and second doses with 95% anti-COPS seroconversion (19 out of 20 mice receiving CVD 1979). However, the COPS-specific antibody titers for mice receiving PBS remained undetectable (data not shown).

### 3.4. Protective Efficacy of Live Attenuated S. Newport CVD 1979 Vaccine

To determine homologous protection, mice were challenged i.p. one month after the third immunization, with *S*. Newport Chile 361. A low (4.3 × 10^5^ CFU) and a high (1.7 × 10^7^ CFU) challenge dose were used. Then, two days post-infection, mice were euthanized, and the bacterial loads in the liver and spleen were determined. The bacterial load (CFU/gram of organ) in the liver of PBS-immunized animals was significantly higher than the bacterial load in animals that received CVD 1979 vaccine (*p* < 0.05) in mice that were administered either the low or high challenge doses. In contrast, bacterial burden in the spleen of the PBS and CVD 1979-vaccinated animals were not significantly different on day 2 post-infection (Figure 1B,C).

**Figure 2:**
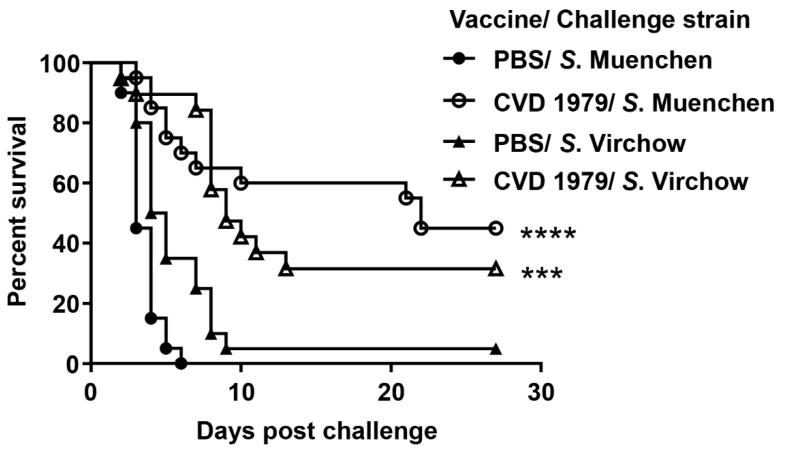
Survival of mice after lethal challenge with *S*. Muenchen (O:8 [C_2_–C_3_]) and *S*. Virchow (O:6,7 [C_1_]). Mice immunized with PBS or CVD 1979, three weeks apart and subsequently challenged intraperitoneally with an LD_100_ of *S*. Muenchen ATCC 8344 and *S*. Virchow Q23. Statistically significant differences in survival curves between the vaccinated and control mice for each challenge strain were determined by log rank test. *** *p* < 0.001; **** *p* < 0.0001.

To determine the cross protective efficacy of CVD 1979 against other O:8 (C_2_–C_3_) and O:6,7 (C_1_) serovars, CVD 1979-immunized (n = 20) and PBS-immunized (n = 20) mice were challenged (i.p.) with 100 × LD_50_ of *S*. Muenchen ATCC 8344 (O:8; C_2_–C_3_) or *S*. Virchow Q23 (O:6,7; C_1_) at 4 weeks post-immunization. All PBS-immunized mice succumbed to infection three to six days after challenge with *S*. Muenchen ATCC 8344, whereas mice immunized with CVD 1979 survived longer (*p* < 0.0001, log-rank test). We calculated a vaccine efficacy of 45% following challenge (Figure 2; *p* = 0.001). In contrast, vaccine CVD 1979 only protected 28% of the mice following challenge with a serogroup O:6,7 (C_1_) serovar, *S*. Virchow (*p* = 0.043). Taken together, our data indicate that the vaccine CVD 1979 was able to reduce the bacterial burden in mice challenged with *S*. Newport and showed moderate protection against lethal challenge with another O:8 (C_2_–C_3_) serovar, *S*. Muenchen, but poor protection against an O:6,7 (C_1_) serovar, *S*. Virchow.

### 3.5. Specificity of CVD 1979 Vaccine-Induced Antibodies

**Figure 3:**
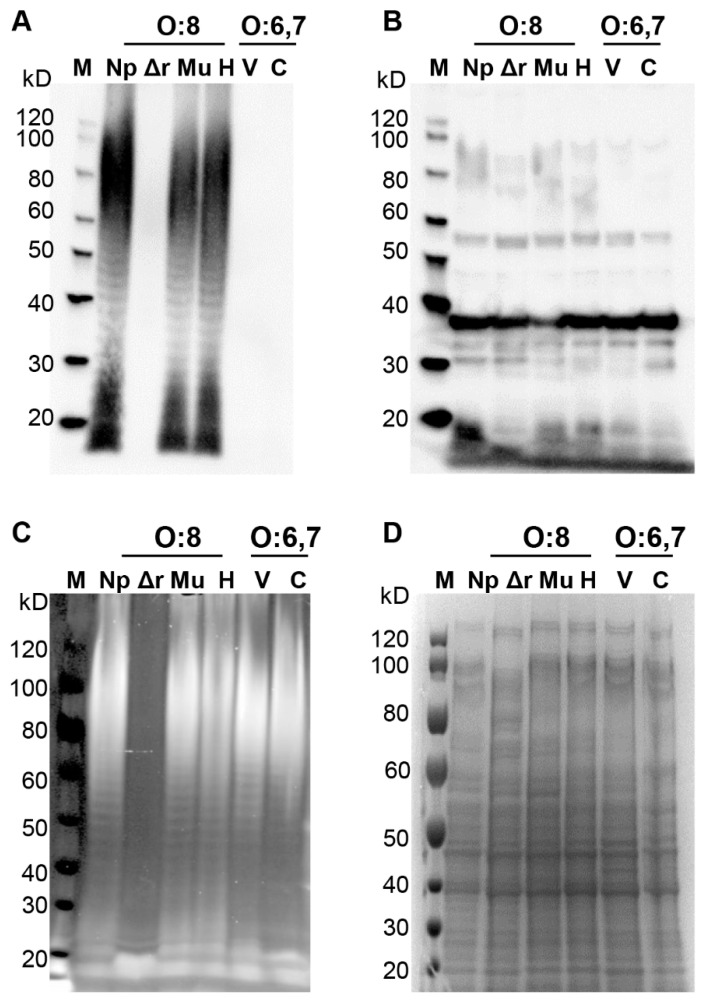
Specificity of antibodies elicited by live attenuated *S*. Newport CVD 1979 vaccine to Lipopolysaccharide (LPS) and whole cell lysates of *Salmonella* strains. LPS and whole cell lysates were extracted from group O:6,7 (C_1_) and O:8 (C_2_–C_3_) serovars. Overnight cultures of *S*. Virchow Q23 (O:6,7), *S*. Choleraesuis var Kunzendorf (O:6,7), *S*. Newport Chile 361 (O:8), *S*. Muenchen ATCC 8344 (O:8), *S*. Hadar 700093 (O:8) and *S*. Newport Δ*rfaL* strains were suspended in PBS and an equal number of cells was harvested to extract LPS and whole cell lysates. After extractions, LPS and cell lysates were separated on a 4–12% NuPAGE^®^ Bis–Tris acrylamide gel. (**A**) Western blot showing reactivity against LPS from group O:8 serovars after probing with pooled sera collected from mice immunized with live attenuated CVD 1979 vaccine. (**B**) Western blot of whole cell lysates probed with pooled immune sera. (**C**) LPS gel stained using the Pro–Q Emerald 300 LPS gel staining kit. (**D**) Whole cell lysates were stained using the gel-code blue Coomassie reagent. M—Marker, Np—Newport, Δr—Newport Δ*rfaL* mutant, Mu—Muenchen, H—Hadar, V—Virchow, C—Choleraesuis var Kunzendorf.

To determine if the reduced protection against the heterologous serogroup was potentially due to anti-COPS antibodies and differences in the O polysaccharide, we assessed the recognition pattern of CVD 1979 vaccine-induced antibodies to LPS and whole cell proteins of O:6,7 (C_1_) and O:8 (C_2_–C_3_) serovars by Western blot. LPS and total crude protein were extracted from *Salmonella* O:6,7 (C_1_) serovars Virchow and Choleraesuis and serogroup O:8 (C_2_–C_3_) serovars Newport, Muenchen and Hadar as well as a *S*. Newport Δ*rfaL* mutant, which does not produce O-side chain polymers. We found that vaccine-induced antibodies can specifically recognize the O antigen in LPS of the O:8 (C_2_–C_3_) serovars (Newport, Muenchen and Hadar) but not the O:6,7 (C_1_) serovars (Virchow and Choleraesuis; Figure 3 and Figure S1). As expected, the *S*. Newport Δ*rfaL* mutant, which lacks O side chain polymers, showed no O antigen bands. Additionally, the CVD 1979 antisera showed similar reactivity to whole cell proteins of O:6,7 and O:8 serovars (Figure 3 and Figure S1). Taken together, the anti-COPS antibodies raised by the live attenuated vaccine are specific to the O antigen of serogroup O:8 and do not show any cross-reactivity with serogroup O:6,7.

### 3.6. Opsonophagocytic Antibody (OPA) Activity

**Figure 4:**
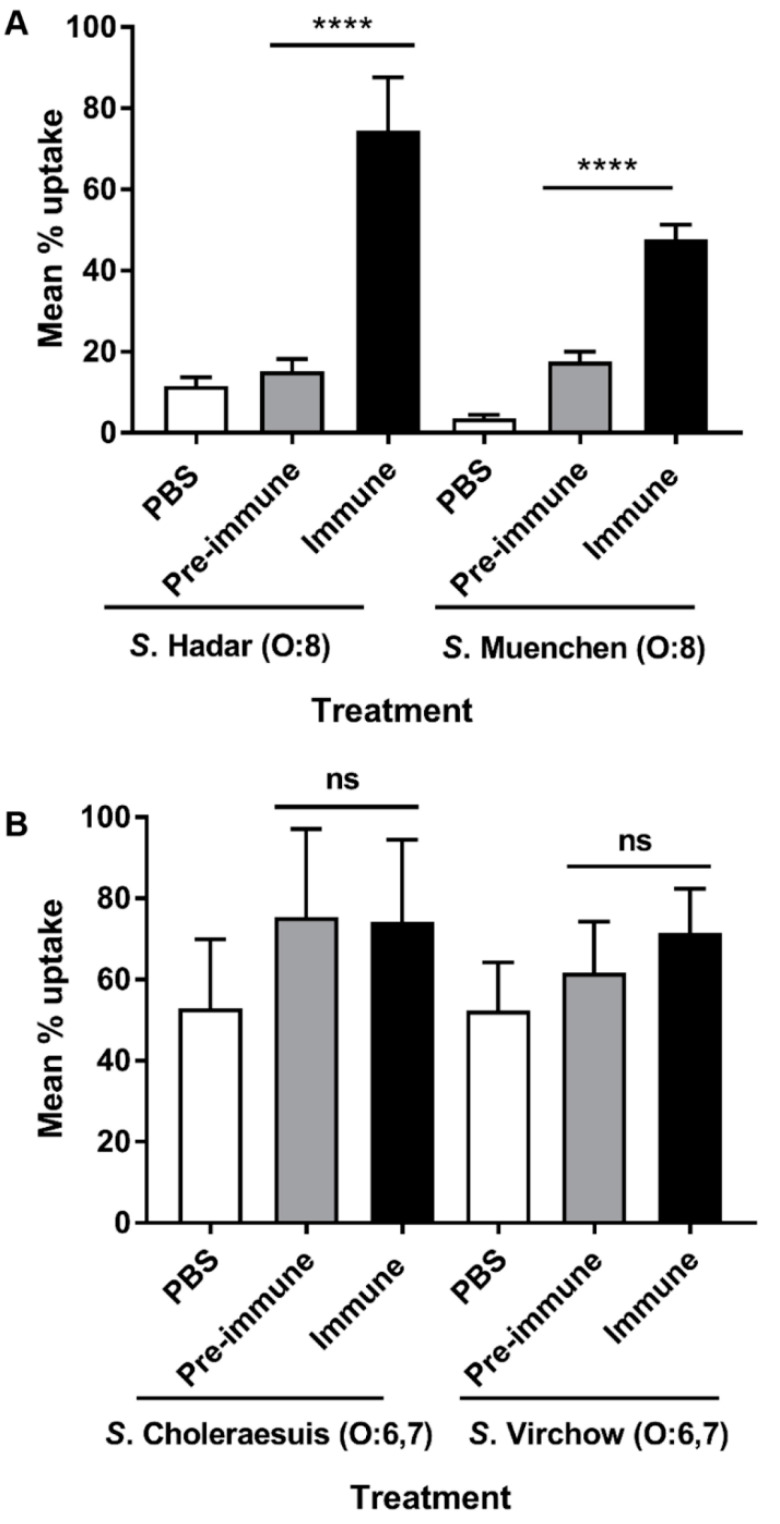
Opsonophagocytic uptake of *Salmonella* group O:6,7 (C_1_) and O:8 (C_2_–C_3_) serovars by J774 macrophages. (**A**) Serogroup O:8 (C_2_–C_3_) strains (*S*. Hadar and *S*. Muenchen) were opsonized with PBS, pre-immune or CVD 1979-immune sera prior to testing for uptake by J774 cells. (**B**) Serogroup O:6,7 (C_1_) strains (*S*. Choleraesuis and *S*. Virchow) were opsonized with PBS, pre-immune or CVD 1979-immune sera prior to testing for uptake by J774 cells. Data shown is the mean of three independent experiments and is presented as mean ± SEM. **** *p* < 0.0001; ns, not significant (unpaired two-tailed Student’s *t*-test).

We next assessed whether vaccine-induced antibodies were capable of opsonizing and promoting phagocytosis of serogroup O:6,7 (C_1_) and O:8 (C_2_–C_3_) serovars by mouse macrophages. We found that sera from mice immunized with CVD 1979 was able to significantly (*p* < 0.0001, Student’s *t*-test) promote phagocytosis of O:8 (C_2_–C_3_) serovars (e.g., *S*. Muenchen and *S*. Hadar) by J774 macrophages in vitro, compared to pre-immunization antisera (Figure 4A). In contrast, the CVD 1979 vaccine-induced sera did not promote phagocytosis of O:6,7 (C_1_) serovars (*S*. Virchow and *S*. Choleraesuis) as compared to the sera of pre-immunized mice (Figure 4B). Our data suggests that antibodies generated by the O:8 (C_2_–C_3_) vaccine can mediate opsonophagocytic uptake of O:8 but not O:6,7 serovars.

**Figure 5:**
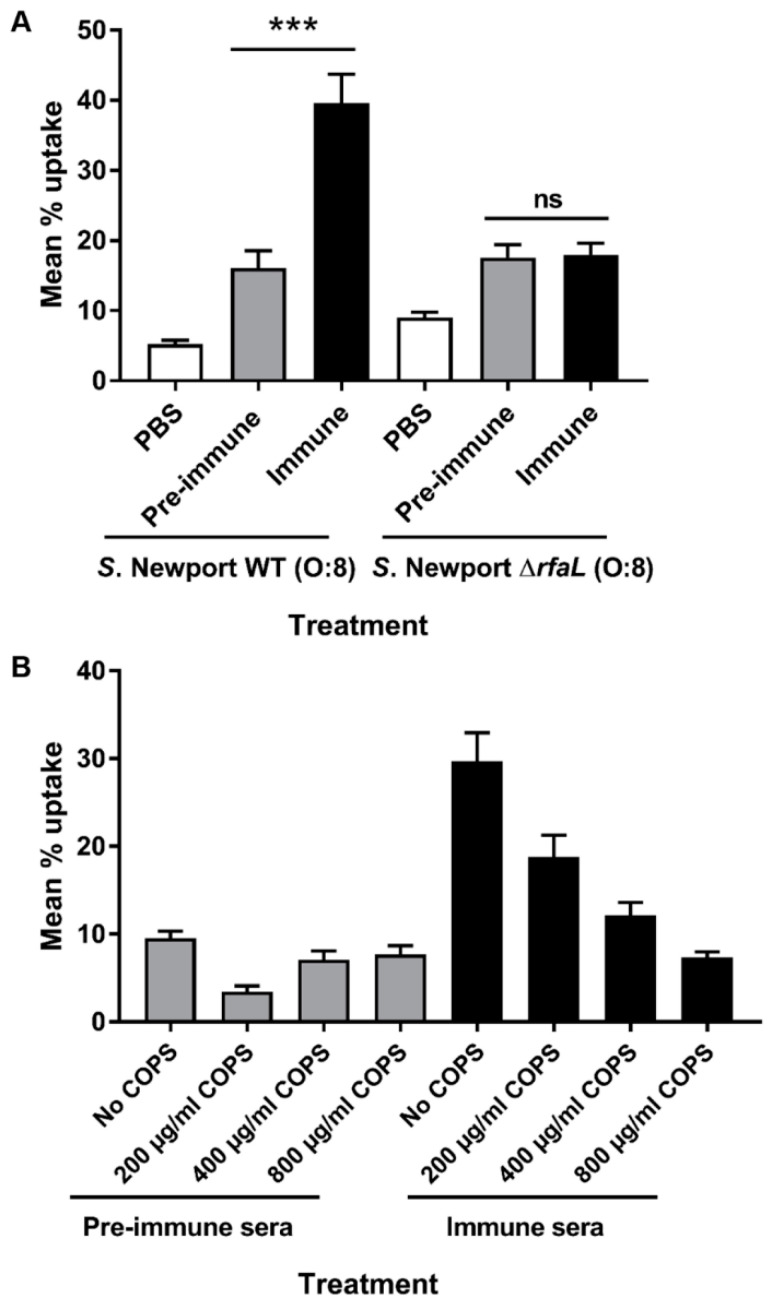
Contribution of anti-LPS antibodies to opsonophagocytic uptake by J774 macrophages. (**A**) *S*. Newport WT and Δ*rfaL* mutant were treated with PBS, pre-immune or CVD 1979-immune sera and uptake by J774 cells was determined. *** *p* <0.001; ns, not significant (two-tailed Student’s *t*-test). (**B**) Serum from CVD 1979-immunized mice was pre-adsorbed with COPS prior to incubation with *S*. Newport WT and then added to J774 cells to determine uptake. Data shown is the mean uptake of three independent experiments and represented as mean ± SEM.

We also determined the opsonophagocytic capability of CVD 1979-induced antibodies for *S*. Newport Δ*rfaL* compared to *S*. Newport WT. We found that the sera from vaccinated mice did not significantly promote phagocytic uptake of the Δ*rfaL* mutant strain suggesting that anti-*O*-polysaccharide antibodies are important for opsonophagocytic uptake of *S*. Newport (Figure 5A). To confirm that the observed OPA activity elicited by CVD 1979 against *S*. Newport was mediated by anti-OPS antibodies, we adsorbed the antisera with increasing amounts of purified COPS and then measured the percent phagocytosis of *S*. Newport following opsonization. We observed a dose-dependent reduction in phagocytosis when COPS-specific antibodies were sequestered, as compared to the unabsorbed sera from immunized mice (Figure 5B).

## 4. Discussion

The goal of this study was to develop an oral live attenuated *Salmonella* Group O:8 (C_2_–C_3_) vaccine that could protect against infections caused by O:6,7 (C_1_) and O:8 (C_2_–C_3_) serovars. Live attenuated vaccines have a variety of advantages over other approaches in that they can induce local mucosal immunity, are economical to produce, and can easily be administered in an oral formulation. Live attenuated vaccine strains can be constructed by incorporating multiple independent defined mutations in bacterial strains using various genetic engineering tools, however, balancing reactogenicity while maintaining, or enhancing, their immunogenicity is challenging [30]. Our initial attempts to develop a live attenuated *S.* Newport vaccine by deleting *guaBA* and *htrA* resulted in an immunogenic vaccine that was well tolerated when administered intraperitoneally to mice but produced some side effects when given perorally. To construct a safer vaccine, we subsequently deleted the *aroA* gene such that there are now three independent attenuating mutations in *S*. Newport and found that the candidate vaccine CVD 1979 was safe and well-tolerated in mice following peroral immunization. As has been shown for other *Salmonella* serovars, we also observed that deletion of *aroA* had an attenuating effect in *S*. Newport as the LD_50_ of the Δ*aroA* mutant in BALB/c mice was found to be 100-fold higher than that of the parental wild-type strain.

Oral immunization of mice with three doses of CVD 1979 produced strong *S*. Newport COPS-specific antibody responses and showed an increase in serum antibody titers after each immunization and reached 95% seroconversion after three immunizations. Importantly, the CVD 1979 vaccine offered protection against colonization of the liver after challenge with wild-type *S*. Newport. We also found that CVD 1979 conferred moderate protection (vaccine efficacy of 45%) against lethal challenge with *S*. Muenchen (another O:8 (C_2_–C_3_) serovar) but poor protection (vaccine efficacy of 28%) against *S*. Virchow which is an O:6,7 (C_1_) serovar. Likewise, we have previously shown that an *S.* Newport COPS:FliC glycoconjugate vaccine was able to confer protection against *S*. Muenchen [18]. Moreover, our in vitro data confirmed that CVD 1979-induced antibodies can only recognize LPS of O:8 (C_2_–C_3_) serovars but not O:6,7 (C_1_) serovars. Both serogroups share the O:6 epitope in their O polysaccharide but we believe that it is a minor epitope that is poorly immunogenic and not a major target of immune responses [18]. We have observed much poorer vaccine efficacy elicited by live attenuated and conjugate *S*. Newport vaccines than for *S*. Typhimurium or *S*. Enteritidis vaccines [21,31]. The pathogenesis of O:8 and O:6,7 strains is very different to other serovars (e.g., Typhimurium) [32,33] and that might play a role in the reduced vaccine efficacy we observed for our *S*. Newport vaccines.

Antibody-mediated bacterial clearance by professional phagocytic cells is an important initial host defense against *Salmonella* infection. Antibodies generated by our live *S*. Newport vaccine CVD 1979 were capable of protecting the host by opsonizing O:8 strains and thus facilitating phagocytosis. However, antibodies generated by the *S*. Newport vaccine were not able to promote uptake of serogroup O:6,7 (C_1_) serovars (*S*. Virchow and *S*. Choleraesuis) or of an *S*. Newport *ΔrfaL* mutant. We also determined the contribution of COPS to opsonophagocytic uptake by preincubating sera with increasing concentrations of purified *S*. Newport COPS antigen. We observed a dose-dependent reduction in phagocytosis when COPS-specific antibodies were sequestered, as compared to the unabsorbed sera from immunized mice. These data suggest that an *S.* Newport live attenuated vaccine elicits opsonophagocytic antibodies that are primarily specific for the O polysaccharide. Importantly, our data suggests that a live attenuated *S*. Newport vaccine cannot mediate cross-protection against lethal challenge with an O:6,7 serovar such as *S*. Virchow and that antibodies generated by the *S*. Newport vaccine cannot mediate opsonophagocytic activity of O:6,7 serovars. The low protection that we observed against challenge with *S*. Virchow might be mediated by cell mediated immunity or IgA or bactericidal antibody.

Our data suggests that separate *Salmonella* O:6,7 and O:8 vaccines might be needed to confer protection against infections caused by serovars of these serogroups. We anticipate that a multivalent vaccine containing live attenuated vaccines that target serogroups O:4, O:9, O:6,7, and O:8 would be needed to provide protection against the majority of NTS serovars that are circulating in humans worldwide.

## 5. Conclusions

In summary, we have developed a safe, oral, live attenuated *S*. Newport vaccine, CVD 1979. Additional experiments are needed to understand the duration of immunity and the mechanism of protection elicited by this vaccine. We have previously developed safe and effective live oral *S*. Typhimurium (O:4) and *S*. Enteritidis (O:9) vaccines. Our next step is to develop a live oral *Salmonella* O:6,7 vaccine which can be combined with the *S*. Typhimurium, *S*. Enteritidis and *S*. Newport vaccines to create a multivalent vaccine effective against O:4, O:9, O:6,7, and O:8 infections.

## Figures and Tables

**Table 1:** *Salmonella* strains used in this study.

Serogroup	Serovar	Strain	Characteristics	Reference
O:8 (C_2_–C_3_)	*S*. Newport	Chile 361	Clinical isolate	[26]
	*S*. Newport	SNE-rfaL	Chile 361 Δ*rfaL*	[19]
	*S*. Newport	SNE-aroA	Chile 361 Δ*aroA*	This work
	*S.* Newport	CDV 1966	Chile 361 Δ*guaBA* Δ*htrA*	[19]
	*S*. Newport	CVD 1979	Chile 361 Δ*guaBA* Δ*htrA* Δ*aroA*	This work
	*S*. Muenchen	ATCC 8344	Wild-type	American Type Culture Collection
	*S*. Hadar	700093	Clinical isolate	[27]
O:6,7 (C_1_)	*S*. Virchow	Q23	Clinical isolate	[28]
	*S*. Choleraesuis var Kunzendorf	CDC-06-0894	Wild-type	U.S. Centers for Disease Control and Prevention

## Data Availability

The data presented in this study are contained within this article and supplementary material.

## Supplementary Materials

The following are available online at https://www.mdpi.com/2076-393X/9/1/57/s1, Figure S1 Unmodified Western bots showing specificity of antibodies elicited by live attenuated *S*. Newport CVD 1979 vaccine to (A) LPS and (B) whole cell lysates.
