# Supplementary material for: Refinement of a Live Attenuated Salmonella enterica Serovar Newport Vaccine with Improved Safety
Source: Vaccines (Basel). 2021 Jan 16;9(1):57. doi: 10.3390/vaccines9010057 (PMC7829832; doi:10.3390/vaccines9010057)
Supplement: Supplementary file 1 [file vaccines-09-00057-s001.pdf]

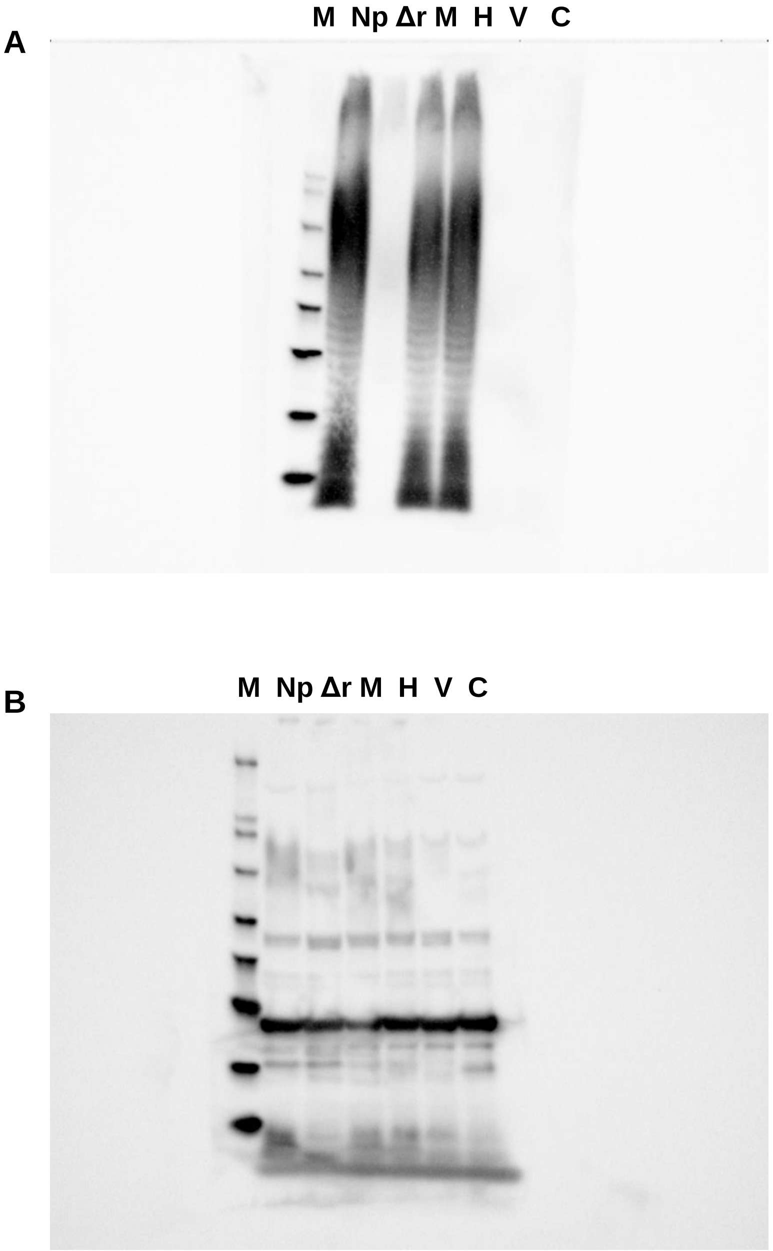

Figure S1. Unmodified Western bots showing specificity of antibodies elicited by live attenuated *S*. Newport CVD 1979 vaccine to A) LPS and B) whole cell lysates.
